# A dedicated C-6 β-hydroxyacyltransferase required for biosynthesis of the glycolipid anchor for Vi antigen capsule in typhoidal *Salmonella*

**DOI:** 10.1016/j.jbc.2022.102520

**Published:** 2022-09-22

**Authors:** S.D. Liston, O.G. Ovchinnikova, M.S. Kimber, C. Whitfield

**Affiliations:** Department of Molecular and Cellular Biology, University of Guelph, Guelph, Ontario, Canada

**Keywords:** Gram-negative bacteria, Salmonella enterica, cell surface, glycoconjugate, glycolipid structure, nuclear magnetic resonance (NMR), acyltransferase, membrane transport, ABC transporter, glycosyltransferase, AcpS, ACP synthase, CPS, capsular polysaccharide, GalNAcA, N-acetylgalactosaminuronic acid, GT, glycosyltransferase, HMBC, heteronuclear multiple bond correlation, HSQC, heteronuclear single quantum coherence, LPLAT, lysophosphatidyl acyltransferase, PIM, phosphatidyl-myo-inositol mannoside, TPR, tetratricopeptide repeat

## Abstract

Vi antigen is an extracellular polysaccharide produced by *Salmonella enterica* Typhi, *Citrobacter freundii*, and some soil bacteria belonging to the Burkholderiales. In *Salmonella* Typhi, Vi-antigen capsule protects the bacterium against host defenses, and the glycan is used in a current glycoconjugate vaccine to protect against typhoid. Vi antigen is a glycolipid assembled in the cytoplasm and translocated to the cell surface by an export complex driven by an ABC transporter. In *Salmonella* Typhi, efficient export and cell-surface retention of the capsule layer depend on a reducing terminal acylated-HexNAc moiety. Although the precise structure and biosynthesis of the acylated terminus has not been resolved, it distinguishes Vi antigen from other known glycolipid substrates for bacterial ABC transporters. The genetic locus for Vi antigen-biosynthesis encodes a single acyltransferase candidate (VexE), which is implicated in the acylation process. Here, we determined the structure of the VexE *in vitro* reaction product by mass spectrometry and NMR spectroscopy to reveal that VexE catalyzes β-hydroxyacyl-ACP dependent acylation of the activated sugar precursor, uridine-5′-diphospho-GlcNAc, at C-6 to form UDP-6-*O*-[β-hydroxymyristoyl]-α-d-GlcNAc. VexE belongs to the lysophosphatidyl acyltransferase family, and comparison of an Alphafold VexE model to solved lysophosphatidyl acyltransferase structures, together with modeling enzyme:substrate complexes, led us to predict an enzyme mechanism. This study provides new insight into Vi terminal structure, offers a new model substrate to investigate the mechanism of glycolipid ABC transporters, and adds biochemical understanding for a novel reaction used in the synthesis of an important bacterial virulence factor.

Bacterial capsules are hydrated cell surface layers, typically formed from high-molecular weight polysaccharides. A wide range of capsular polysaccharide (CPS) structures are known, and their functions are equally diverse (1). Capsules are common in some species in the family Enterobacteriaceae, with pathogens such as *Escherichia coli* and *Klebsiella pneumoniae* each producing many antigenically distinct CPS with differing structures (1, 2, 3). In contrast, Vi antigen is the only capsule type known in *Salmonella* (3). Vi antigen is associated with resistance to the host innate immune response and is produced by typhoidal isolates but not by representatives of serovars that cause gastroenteritis (reviewed in (4)). Vi antigen is also produced by isolates of *Citrobacter freundii* (5), and the conserved Vi antigen locus is also found in some soil bacteria belonging to the *Bordetella* and *Achromobacter* genera, where the biological role of the polysaccharide is unknown (6). Purified Vi antigen is used in current typhoid vaccines (7) and protein-Vi antigen conjugate vaccines have been developed (8, 9, 10, 11). Recent studies have described refined conjugate compositions to obtain an optimized immunological response (12).

Vi antigen is a linear polymer of α-1,4-linked 2-acetamido-2-deoxygalacturonic acid (*N*-acetylgalactosaminuronic acid; GalNAcA) residues, which are *O*-acetylated nonstoichiometrically at C-3 (5). Assembly of the Vi-antigen capsule uses an “ABC transporter-dependent” strategy, where the polysaccharide is polymerized in the cytoplasm and exported across the bacterial cell envelope by an export complex comprised of the ABC transporter, a periplasmic adapter protein, and an outer membrane translocon (1, 3). Similar export systems are employed in production of many CPS structures in diverse Gram-negative bacteria. However, in other known examples, the polysaccharides are built on the nonreducing terminus of a conserved acceptor glycolipid composed of a short oligosaccharide of β-linked 3-deoxy-D-*manno*-oct-2-ulosonic acid residues linked to phosphatidylglycerol (13, 14, 15). Vi antigen is the sole known outlier and *S*. Typhi lacks the genes necessary for synthesis of this 3-deoxy-D-*manno*-oct-2-ulosonic acid-containing glycolipid acceptor. Instead, the Vi terminus possesses a reducing terminal HexNAc residue modified with two hydroxylated acyl chains, which is required for efficient export and anchoring the glycan on the cell surface (6). The glycolipid terminus was identified by mass spectrometry of hydrophobic glycoconjugates liberated from high molecular weight *S*. Typhi Vi antigen by a Vi antigen depolymerase (VexL) from *Achromobacter denitrificans* (a Vi antigen-producer) (6). Unfortunately, the fragmentation pattern in the mass spectrum did not definitively establish the acyl linkage position nor could it provide insight into the configuration of the HexNAc residue. Subsequent attempts to purify sufficient glycolipid from bacterial cultures for NMR spectroscopy have been unsuccessful due to low yield; terminal residues represent a small fraction of the total mass of purified long-chain polysaccharides and there is inevitably a substantial loss of material during the purification process.

The Vi-antigen capsule assembly system is an important prototype for understanding the assembly and export of bacterial glycolipids. It remains unclear whether other related variants of this assembly strategy exist but resolving the structure of the Vi antigen terminal lipid may provide hallmarks for other systems. Furthermore, the terminal lipid structure has implications for understanding the guiding principles for recognition and export of glycolipid substrates by ABC transporters, which may have implications in bioengineering commercial polymer production. Only one candidate acyltransferase enzyme (VexE) is encoded by the Vi antigen biosynthesis genetic locus. Here, we describe the biochemical activity of VexE using an *in vitro* strategy and identify a new glycolipid structure.

## Results

### VexE is an acyl carrier protein–dependent UDP-GlcNAc C-6 β-hydroxyacyltransferase

**Figure 1:**
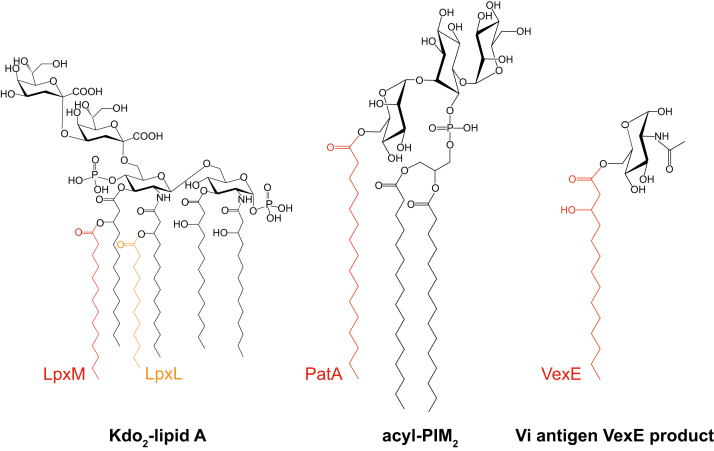
**Bacterial glycolipid substrates whose synthesis involves LPLAT proteins.** LpxL and LpxM are secondary lauroyl and myristoyl transferases, respectively, from Kdo_2_-lipid A biosynthesis. PatA is an acyltransferase that palmitoylates PI-Man_2_. PIMs are membrane glycolipids produced by *Mycobacteria*, for example. The product of VexE in Vi-antigen biosynthesis is described in this study. LPLAT, lysophosphatidyl acyltransferase; PIM, phosphatidyl-myo-inositol mannoside.

Initial bioinformatic analyses identified a putative C-terminal lysophosphatidyl acyltransferase (LPLAT) domain in VexE homologs from *S.* Typhi and *A*. *denitrificans* (6). Structures are available for two bacterial LPLAT representatives with different functions; *Acinetobacter baumannii* LpxM (16) and *Mycobacterium smegmatis* PatA (17, 18, 19). LpxM is one of two secondary acyltransferases involved in the biosynthesis of LPS lipid A by the Raetz pathway, which is conserved in most Gram-negative bacteria (reviewed in (20)). LpxM myristoylates β-hydroxyacyl groups on the 3ʹ acyl chain attached to the distal glucosamine residue in the lipid A backbone (Fig. 1). In contrast, PatA is a “primary” acyltransferase that acylates a mannose residue at C-6 in the biosynthesis of phosphatidyl-*myo*-inositol mannosides (PIMs), which are a family of abundant and important cell envelope membrane glycolipids produced by Mycobacteria (21) (Fig. 1).

Synthesis of Vi antigen requires a single-site UDP-GalNAcA–dependent glycosyltransferase (GT) enzyme (TviE), which belongs to the GT4 family (22). TviE operates in the absence of a polyprenol carrier lipid, similar to processive single-site GTs involved in bacterial cellulose and hyaluronic acid biosynthesis. Cellulose synthase initiates polymerization using a UDP-glucose molecule (23) and the *Chlorella* virus hyaluronan synthase primes the catalytic process by hydrolysis of the UDP-GlcNAc (24). Both synthases extend the glycan at the nonreducing terminus. Although mechanistically different, Streptococcal class I hyaluronan synthases also build on a UDP-linked primer (25, 26). The reducing terminal residue of Vi antigen possesses β-hydroxy-fatty acids (6), like lipid A, where the Raetz pathway involves sequential acylation of UDP-linked intermediates (beginning with UDP-GlcNAc) (20). Collectively, this information led to the hypothesis that the Vi antigen acylation reaction might proceed by modification of a “housekeeping” UDP-HexNAc donor, with UDP-GlcNAc being the likely candidate. Acyltransferase enzymes use activated acyl donors, which are generated by covalent thiol linkage to CoA or holo-acyl carrier protein (ACP). While PatA uses CoA-activated donors (17, 19), the preferred donors for secondary acyltransferases in lipid A biosynthesis (LpxL and LpxM) are acyl-ACP derivatives (16, 27, 28). Therefore, as a first step toward biochemical characterization of VexE, its potential acyltransferase activity was investigated in an *in vitro* reaction mixture containing UDP-GlcNAc and acyl-ACP donors. Attempts to produce soluble *S.* Typhi VexE in *E. coli* were unsuccessful (Fig. S1), but the soluble protein was obtained with the *A. denitrificans* homolog, so it was used in biochemical studies.

Since donors for β-hydroxyacyl chains are not commercially available, a series of acyl-ACP donors were synthesized enzymatically. ACP and holo-ACP synthase (AcpS) were coexpressed and purified from *E. coli*; AcpS transfers a phosphopantetheine functional group to ACP, generating the holo-enzyme (holo-ACP) (29, 30). Purification of holo-ACP followed a scheme adapted from previously published methods (31) and employed IMAC and AEC chromatography (Fig. S2*A*), together with covalent chromatography using a resin that binds free sulfhydryl residues. This protocol separates holo-ACP from the acyl- and apo-protein, which do not contain a free thiol group. Purified soluble acyl-ACP synthetase (AasS) from *Vibrio harveyi* (Fig. S2*B*) was then used to load holo-ACP with fatty acids *in vitro* (31, 32). These loading reactions were monitored by SDS-free Tris-glycine PAGE in the presence of 2.5 M urea (Fig. S2*C*). Under these conditions, electrophoretic mobility of purified holo-ACP increases dramatically when loaded with its fatty acid cargo (33). Based on shifts in electrophoretic mobility of the acyl-ACP product, AasS efficiently loaded holo-ACP with acyl and β-hydroxyacyl chains up to 16 carbons in length (Fig. S2*D*). The presence of two bands in PAGE of the β-hydroxyacyl-ACPs is presumed to reflect the racemic mixture of DL-3-hydroxy-fatty acids used.

**Figure 2:**
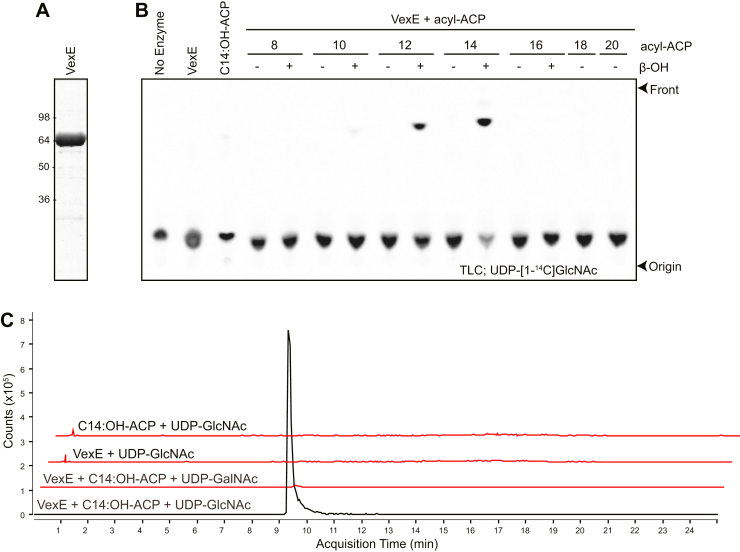
**VexE is an acyl-ACP–dependent UDP-GlcNAc β-hydroxyacyltransferase.***A*, SDS-PAGE of purified VexE-His_6_. *B*, VexE acylates UDP-GlcNAc *in vitro*, with an absolute requirement for β-hydroxylation of the acyl donor, and preference for 12- to 14-carbon chain lengths. The panel shows TLC analysis of acyltransferase reactions containing purified VexE, UDP-[1-^14^C]-GlcNAc, and indicated purified acyl-ACP donor. *C*, acceptor preference for VexE. The traces depict extracted ion chromatograms of HPLC-MS/MS separation of VexE reaction products using the preferred substrate UDP-GlcNAc and the (non-substrate) UDP-GalNAc. The *vertical axis* represents counts in MS^2^ for the diagnostic [M-H]^−^ ion at *m*/*z* 526.242, which is the expected *m*/*z* value for β-hydroxymyristoyl-HexNAc-1-phosphate. No product was detected unless VexE, acyl-ACP, and UDP-GlcNAc were all present.

Purified VexE (Fig. 2*A*) directly modified UDP-[1-^14^C]-GlcNAc to create a single new species, detected as a faster migrating product in TLC (Fig. 2*B*). Activity was dependent on β-hydroxylation of the donor and showed a clear chain-length preference for C14 > C12 > C10 under these conditions, consistent with the acyl chains predicted in the natural product by MS data (6). Because the configuration of the terminal hexose residue in the glycolipid terminus was unknown, acylation reactions were also performed with unlabeled UDP-GalNAc and β-hydroxymyristoyl-ACP with products analyzed using HPLC-MS (Fig. 2*C*). Using UDP-GlcNAc as the acceptor, a product with a mass corresponding to UDP-(β-hydroxymyristoyl)-GlcNAc was detected, but no product was synthesized with UDP-GalNAc. These data indicate that VexE is an acyl-ACP–dependent β-hydroxyacyltransferase and support the conclusion that the HexNAc residue in the authentic glycolipid terminus possesses the *gluco* configuration.

### Structure of the *in vitro* reaction product

**Figure 3:**
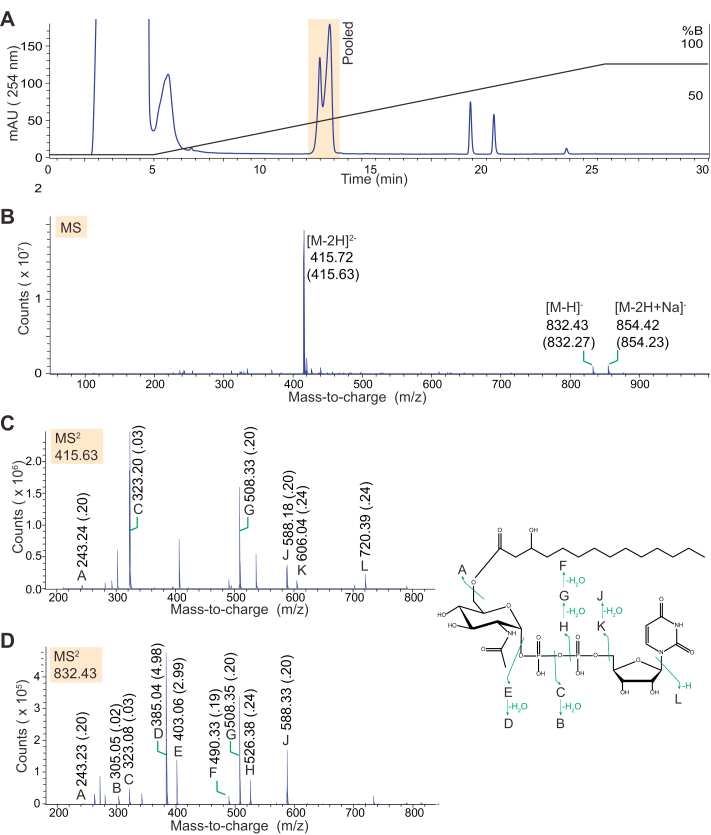
**Mass spectrometry analysis of the VexE reaction product.***A*, HPLC separation of VexE acylation reactions employing UDP-GlcNAc and β-hydroxymyristoyl (C14)-ACP substrates, monitored by spectrophotometry at 254 nm. *B*, negative-mode direct-infusion ESI-MS spectrum of the pooled and concentrated fractions indicated in (*A*). *C*, MS^2^ fragmentation of the ion at *m*/*z* 415.63, which corresponds to doubly-charged UDP-β-hydroxymyristoyl-GlcNAc. *D*, MS^2^ fragmentation of the ion at *m*/*z* 832.43, which corresponds to singly-charged UDP-β-hydroxymyristoyl-GlcNAc. Expected *m*/*z* are indicated in parentheses adjacent to those observed. Fragmentation products are illustrated by *green arrows*. The linkage position of the acyl chain reflects NMR data collected from this material and was not informed by MS.

To definitively establish the structure of the reaction product, scaled-up acylation reactions containing VexE, β-hydroxymyristoyl-ACP, and nonradioactive UDP-GlcNAc were performed, and the hydrophobic product was purified by HPLC (Fig. 3*A*). The mass spectrum of the reaction mixture and mass spectrometry fragments were consistent with the product being UDP-(β-hydroxymyristoyl)-GlcNAc (Fig. 3*B*). However, as observed with the native glycolipid terminus (6), MS^2^ fragmentation did not establish the linkage position of the acyl chain (Fig. 3, *C* and *D*). This gap in the structural information was resolved by NMR spectroscopy.

**Figure 4:**
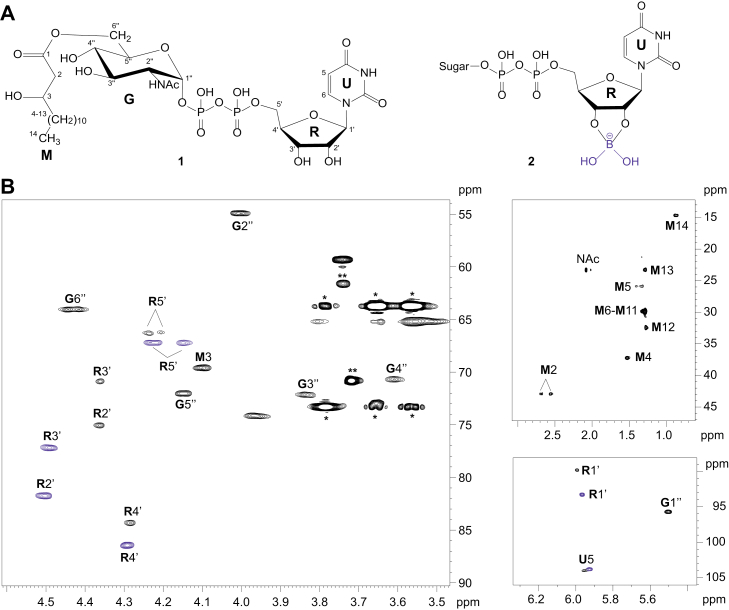
**NMR spectroscopy of the VexE product.***A*, the structure of the main VexE product (1) and borate diol monoester of UDP-sugar (2). In the samples, a mixture of borate diol monoester and two diastereomeric diesters is expected; however, our NMR data could not distinguish between these complexes. *B*, selected parts of the ^1^H,^13^C HSQC spectrum of VexE product. C/H pairs of moieties are labeled as follows: U, uracil; R, ribose, G, glucosamine, M, β-hydroxymyristoyl group. *Blue signals* denote uracil and ribose participating in the borate complex. Signals for contaminating glycerol (∗) and PEG (∗∗) are indicated. HSQC, heteronuclear single quantum coherence.

The purified product of VexE was analyzed by ^1^H, ^13^C, and ^31^P NMR spectroscopy (Fig. 4). The presence of the β-hydroxymyristoyl moiety was evident from the characteristic triplet at δ_H_ 0.87 for the terminal methyl protons (H-14), which appeared to have roughly the same integral intensity as the signal for the *N*-acetyl group of GlcNAc (δ_H_ 2.08). The heteronuclear ^1^H, ^13^C single quantum coherence (HSQC) spectrum contained additional characteristic signals (at δ_H_/δ_C_ 2.55/43.0 and 2.69/43.0) for α-methylene protons (H-2) adjacent to the carbonyl group (Fig. 4*B*). The ^13^C NMR chemical shift for the carbonyl carbon (C-1) was obtained from heteronuclear multiple bond correlation (HMBC) spectroscopy data. The remaining signals for the β-hydroxymyristoyl group were assigned based on 2D COSY, TOCSY, HSQC, and HMBC experiments, and by comparison with published data (34, 35). ^1^H and ^13^C NMR chemical shifts are summarized in Tables 1 and 2, respectively.

**Table 1:** ^1^H NMR data (25 °C, D_2_O)

Moiety	VexE product (free, minor)^a^	VexE product−borate complex^a^	UDP-GlcNAc	UDP-GlcNAc−borate complex
Uracil (**U**)				
H-5	5.95	5.93	5.97	5.93
H-6	7.93	7.81	7.97	7.82
Ribose (**R**)				
H-1’	5.99	5.96	5.99	5.97
H-2’	4.36	4.50	4.38	4.51
H-3’	4.36	4.49	4.37	4.49
H-4’	4.28	4.29	4.29	4.29
H-5’	4.21, 4.23	4.14, 4.22	4.20, 4.25	4.14, 4.22

Pyranose	residue **G**	residue **H**		

H-1’’	5.50	5.56	5.52	5.52
H-2’’	4.00	4.20	4.00	4.00
H-3’’	3.83	5.23	3.81	3.82
H-4’’	3.61	3.76	3.56	3.56
H-5’’	4.15	4.03	3.94	3.94
H-6’’	4.41, 4.44	3.82, 3.87	3.81, 3.86	3.81, 3.86
Acetyl				
CH_3_	2.08	2.08	2.08	2.08
β-hydroxymyristoyl (**M**)				
H-2	2.55, 2.69			
H-3	4.10			
H-4	1.52			
H-5	1.33, 1.40			
H-6−H-11	1.27−1.33			
H-12	1.27			
H-13	1.28			
H-14	0.87			

a One set of signals was observed for GlcNAc6Acyl (G), GlcNAc3Acyl (H), and β-hydroxymyristoyl moieties.

**Table 2:** ^13^C NMR data (25 °C, D_2_O)

Moiety	VexE product (free, minor)^a^	VexE product−borate complex^a^	UDP-GlcNAc^b^	UDP-GlcNAc−borate complex^b^
Uracil (**U**)				
C-2	154.5	153.6		
C-4	169.4	168.6		
C-5	104.0	103.8	103.9	103.8
C-6	142.6	143.9	142.9	144.0
Ribose (**R**)				
C-1’	89.8	93.3	89.7	93.3
C-2’	75.1	81.8	75.1	81.7
C-3’	70.9	77.2	70.9	77.2
C-4’	84.3	86.4	84.5	86.5
C-5’	66.3	67.2	66.2	67.3

Pyranose	residue **G**	residue **H**		

C-1’’	95.7	95.6	95.8	95.8
C-2’’	54.9	53.1	55.0	55.0
C-3’’	72.2	75.1	72.2	72.2
C-4’’	70.7	68.6	70.8	70.8
C-5’’	72.1	74.1	74.3	74.3
C-6’’	64.1	61.4	61.6	61.6
Acetyl				
CH_3_	23.4	23.4	23.4	23.4
CO	176.0	176.0		
β-hydroxymyristoyl (**M**)				
C-1	175.2			
C-2	43.0			
C-3	69.6			
C-4	37.3			
C-5	25.9			
C-6−C-11	30.0			
C-12	32.5			
C-13	23.3			
C-14	14.7			

a One set of signals was observed for GlcNAc6Acyl (G), GlcNAc3Acyl (H), and β-hydroxymyristoyl moieties.

b Data from HSQC experiment.

The anomeric signal (H-1’’/C-1’’) in the HSQC spectrum at δ 5.50/95.7 was employed as a starting point to assign signals for the GlcNAc residue (residue **G**). A small *J*_1’’,2’’_ coupling constant of ∼3 Hz and *J*_1’’,P_ of 7.2 Hz support α-d-configuration of the GlcNAc residue. The TOCSY spectrum demonstrated correlations between H-1’’ and H-2’’−H-6’’ protons, which is characteristic for sugars with the *gluco* configuration, and correlations traced in the COSY spectrum were used to distinguish protons within the spin system. ^13^C NMR chemical shifts for the GlcNAc residue were obtained from HSQC and HMBC spectra. Since a three-bond correlation was not observed between C-1 of the β-hydroxymyristoyl group and any of the GlcNAc protons in the HMBC spectrum, the position of acyl substituent at C-6 was inferred from ^1^H and ^13^C chemical shifts of the GlcNAc residue. Acylation of a sugar hydroxyl group results in downfield displacement of the signal for the proton at the acylation site (36). C-6 acylation is reflected by the low field position observed for H-6’’a,b at δ 4.41 and 4.44, compared with their position at δ 3.81 and 3.86 in UDP-GlcNAc (Table 1). The location of the *O*-acyl substituent at position 6 was confirmed by a downfield shift (+2.5 ppm) of the signal for C-6’’ and an upfield shift (−2.2 ppm) of the signal for the neighboring carbon C-5’’ compared with their position in UDP-GlcNAc (Table 2).

The NMR spectra also contained minor series of signals that were assigned to unsubstituted GlcNAc (residue **F**) and GlcNAc O-acylated at position 3 (residue **H**) (Figs. S3 and S4). The location of the acyl group in the latter was evident from low field resonance of **H** H-3’’ at δ 5.23, as well as an upfield shift of the signals for the neighboring carbons **H** C-2’’ and C-4’’ (Tables 1 and 2). Based on integrated intensity of the signals for **G** H-1 and **H** H-3 in ^1^H NMR spectrum, the 3-acylated compound comprises less then 10% of the products with the remainder being the major 6-acylated derivative. We suspect this may be an artefact of the *in vitro* system, but it can only be resolved unequivocally with a complete structure of the native glycolipid. Molecules simultaneously acylated at both positions were not observed in NMR spectra or in the mass spectra of the *in vitro* reaction mixture prior to purification.

The remaining signals for the uracil and ribose moieties of the VexE product were split. The minor signal series was virtually identical to published values for uracil and ribose signals in UDP-GlcNAc, whereas the major series differed from UDP-GlcNAc, free UDP, and UMP. The ^31^P spectrum contained two signals for the diphosphate group at δ −10.8 and −12.7, which were assigned based on correlations with ribose H-5’, GlcNAc H-1’’ and H-2’’ in the ^1^H,^31^P HMBC spectrum. Both ^31^P signals were split, which reflects structural heterogeneity in the uridine moiety. Ribose carbons C-1’−C-5’ in the major signal series all resonated downfield of those in UDP-GlcNAc, with the largest difference being for C-2’ and C-3’ (∼6.5 ppm downfield shift). This was indicative of a diester borate complex. A similar change in chemical shift upon borate esterification of 6 to 7 ppm was reported for C-2 and C-3 of apiofuranosides (37), which possess the same relative stereochemistry at C-2 and C-3 as ribose. The formation of borate complexes with *cis*-diol groups of ribose residues has been reported (38), but NMR data for borate–nucleotide complexes are not available. To confirm the presence of a borate complex with the VexE product, a UDP-GlcNAc−borate complex was prepared by mixing equimolar amounts of boric acid and UDP-GlcNAc in D_2_O and adjusting pH to 8, as described for apiofuranosides (37). ^1^H NMR and HSQC spectra of this mixture contained an additional set of signals, when compared to UDP-GlcNAc alone. The ratio of free to borate-esterified UDP-GlcNAc in this sample was 5.6:1, based on the ratio of the integral intensities of the uracil ^1^H-6 signals. The ^1^H and ^13^C NMR chemical shifts of the uracil and ribose in the UDP-GlcNAc−borate complex were essentially identical to those observed in the major signal series in the HSQC spectrum of the VexE product (Tables 1 and 2).

The VexE product began to precipitate from solution after a week of storage at 4 °C in NMR ampule, likely due to cleavage of the hydroxymyristoyl group. The borate complex was disrupted by lowering the pH of this sample to 1, resulting in the disappearance of the corresponding signals in the HSQC spectrum and increased intensity of the nonesterified ribose signals. In addition, the signals for GlcNAc **G** H-6’’a,b shifted upfield to δ 3.80 and 3.86, indicating that position 6 was no longer *O*-acylated. This borate complex is most likely an *in vitro* artefact reflecting the low sample concentration and the use of borosilicate glass. Since it has no bearing on the conclusions concerning the product structure, it was not pursued further.

### Structure-sequence-function relationships of VexE

**Figure 5:**
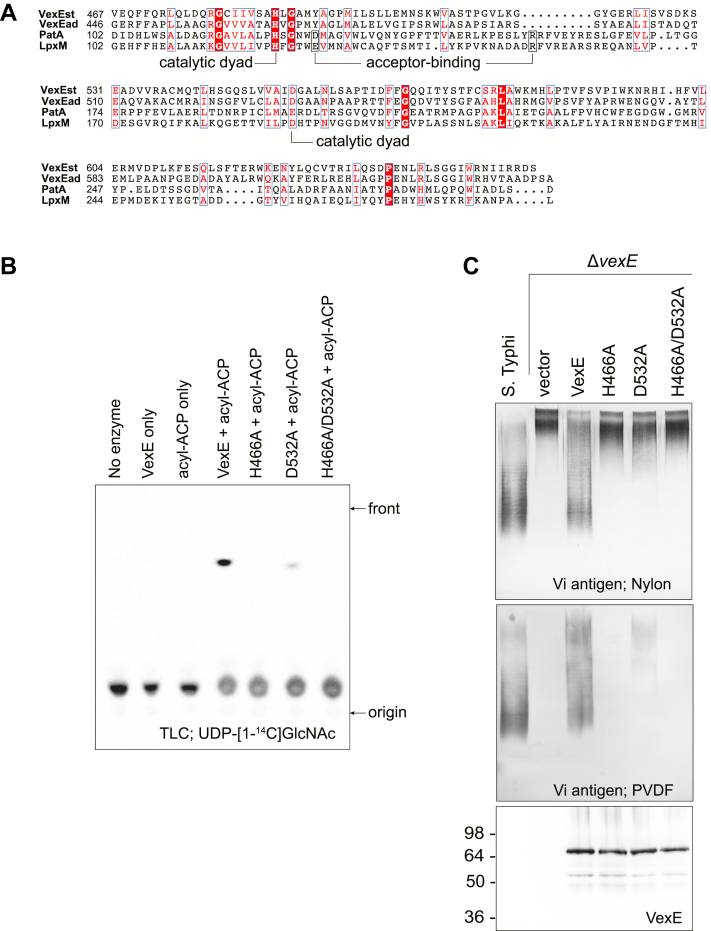
**Site-directed mutagenesis of predicted catalytic residues of VexE.***A*, partial sequence alignment showing the HX_4_D/E motif and other relevant residues. *B*, *in vitro* product formation was dependent on catalytically active VexE. No product was evident for purified VexE H466A or VexE H466A/D532A. *C*, as shown previously, WT Vi antigen bound both PVDF and Nylon membranes, whereas Vi antigen produced by a Δ*vexE* mutant possessed increased apparent molecular weight and bound only to Nylon (6). Expression of WT VexE restores PVDF binding and lowers apparent molecular weight of the Vi antigen. VexE D532A restored some PVDF-binding, whereas VexE H466A and VexE H466A D532A did not. The figure depicts western immunoblots of whole-cell lysates prepared from *Salmonella* Typhi or *S*. Typhi Δ*vexE*–expressing plasmid-encoded VexE or site-directed mutant derivatives. Immunoblots were probed with Vi antigen– or pentahistidine-specific antibodies.

LPLAT proteins typically contain a characteristic H(X)_4_D/E motif (16, 17, 18, 39) (Fig. 5*A*). In lysophosphatidic acid acyltransferases, the histidine and Asp/Glu form a hydrogen bond and are proposed to act in concert to activate glycerol as a nucleophile (39). In PatA and LpxL, the corresponding Glu is positioned some 8.5 Å away from the His and instead appears to interact with a conserved arginine to help bind the acyl substrate (16). Molecular dynamics simulations of PatA (based on costructures with substrates/products) suggest that the conserved His instead coordinates with a topologically distinct acidic residue (here Glu200) to directly deprotonate the C-6 hydroxyl of the mannose acceptor, which then attacks the acyl-thioester donor (18). A histidine to alanine replacement at this position in LpxL reduces its activity by >1000-fold (28) and explains our previous observation with *S.* Typhi that a *vexE*-deletion or a VexE^H466A^ mutation both resulted in impaired export of Vi antigen and an inability to retain the exported polysaccharide on the cell surface (6). This phenotype was correlated with altered physical properties of the Vi antigen, evident in an inability of the polymer to bind to PVDF blotting membrane, while binding to nylon was unaffected (recapitulated in Fig. 5*C*). These results were consistent with altered acylation of Vi antigen, and this was confirmed here by *in vitro* reactions showing the VexE^H466A^ mutant retained no detectable activity (Fig. 5*B*). Interestingly, the usually conserved Asp/Glu from the H(X)_4_D/E motif is replaced by a tyrosine in VexE; this residue tolerated mutagenesis to phenylalanine (6). To add support for this predicted catalytic site organization, the Vi-antigen phenotype conferred by a VexE^D532A^ (where VexE^D532^ is homologous to PatA^E200^) was assessed. The *in vitro* acyltransferase activity of the mutant was substantially reduced, as was the PVDF-binding capacity of the corresponding Vi antigen (Fig. 5, *B* and *C*). As expected, the H466A D532A double mutant was inactive. Collectively, these data are consistent with the conclusion that VexE contains a catalytically competent active site, despite possession of a tyrosine at the sixth position in the canonical LPLAT H(X)_4_D/E motif.

To better understand how VexE functions, the AlphaFold model of *S.* Typhi VexE structure was retrieved from the EBI database (entry AF-P43112-F1-model_v2) (40, 41) and analyzed. This structural model appears to be reliably predicted, with a predicted local distance difference test score (pLDDT) >90 for most of the structure (including the functionally important regions) and the predicted aligned error indicates confidence in the relative placement of the two domains (Fig. S5, *A* and *B*). The protein appears to be monomeric, based on the absence of features associated with oligomeric interaction sites (exposed nonpolar patches or extended conserved surfaces outside of functional sites). There are also no extended hydrophobic patches that seem likely to associate with the membrane, consistent with the observation that VexE from *A. denitrificans* is a soluble protein.

**Figure 6:**
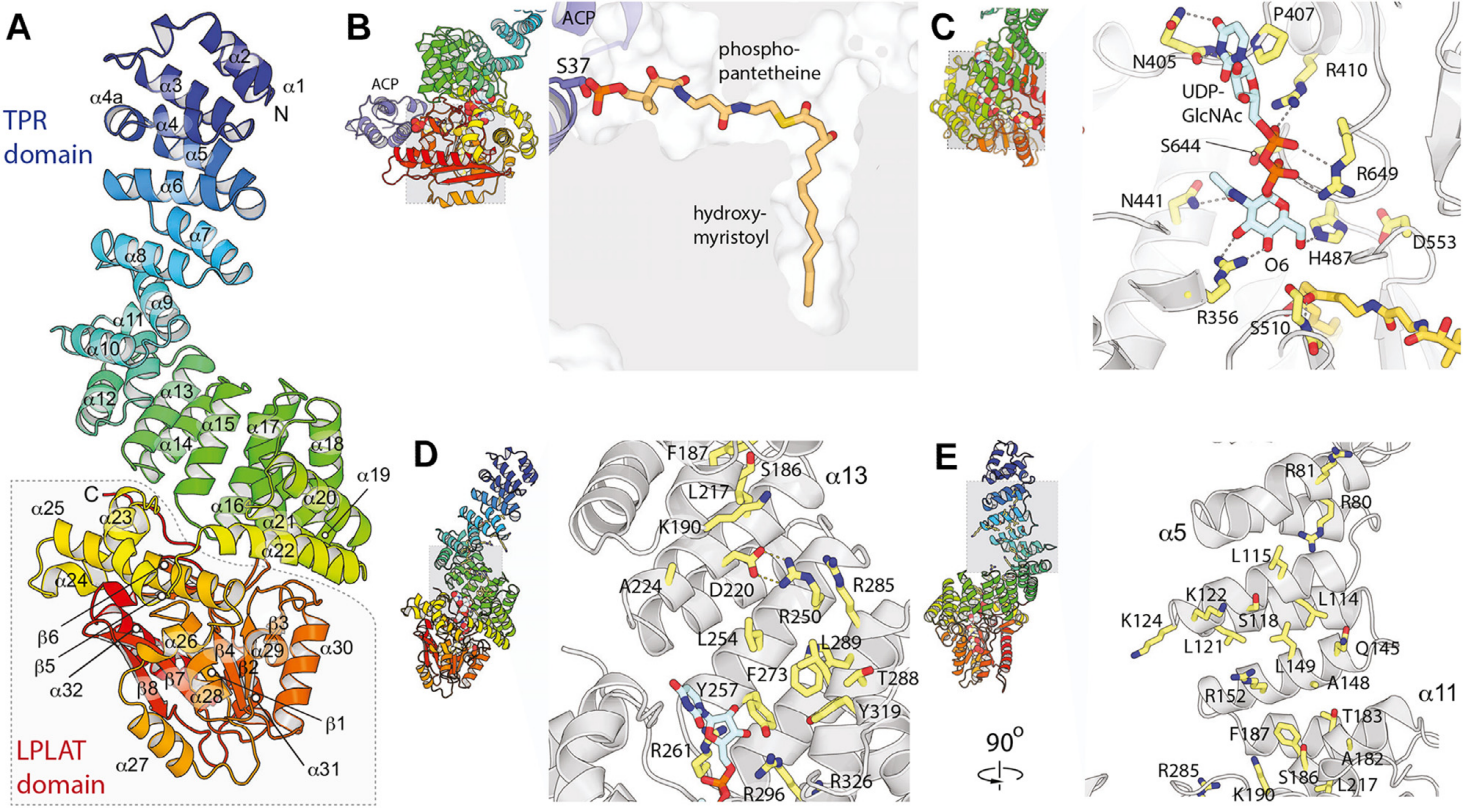
**AlphaFold structure and substrate models of *Salmonella* Typhi VexE.***A*, overall structure of *S.* Typhi VexE. The catalytic LPLAT domain is outlined with the box and α22 represents the end of the TPR domain. *B*, slice through the VexE substrate complex model, showing the organization of the β-hydroxymyristoyl-phosphpantetheine–binding tunnel. *C*, details of the proposed UDP-GlcNAc–binding site and catalytic site. *D*, details of the concave side of the TPR superhelix close to the LPLAT domain. Note the formation of a hydrophobic groove flanked by basic groups. D^220^ and R^250^ form a salt bridge, they only present nonpolar motifs to any potential interaction partners and therefore function as part of the hydrophobic groove despite their nominally polar natures. *E*, distal portion of the same TPR surface. The regions expanded in (*C*–*E*) are highlighted by the *gray boxes* on the adjacent structural thumbnail images. LPLAT, lysophosphatidyl acyltransferase; TPR, tetratricopeptide repeat.

The N-terminal domain of VexE (residues 1–385) is built as a series of 11 tetratricopeptide repeat (TPR) repeats, while the C-terminal domain (residues 386–686) of VexE adopts a fold similar to known LPLAT proteins (Fig. 6*A*). This LPLAT domain is built around a six-stranded, highly curved, predominantly parallel β-sheet (with topology 3,2,4,1,7,-8), with helices packing on both faces. A two-stranded β-hairpin along with four additional α-helices extend this domain along one face, interacting with the TPR domain and forming the extended active site. The last four TPR repeats pack onto the LPLAT domain, while the first seven form a long super-helical extension. Searching the protein data bank with the structure similarity search tool DALI (42) suggests that no currently structurally characterized proteins share the observed TPR-LPLAT domain combination. Using the LPLAT domain as a search model reveals that the two closest homologs are PatA (PDB: 5ft2) and LpxM (PDB: 5knk) with Z-scores of 18.2 and 17.7, respectively (Table S1). Note that these structures have r.m.s.d. values of 3.2 and 3.4 Å, and sequence identities of 10 and 15%, indicating that VexE is divergent from PatA and LpxM. The only other significant structural homolog is glycerol-3-phosphate acyltransferase (PDB: 5kym and 1k30), with Z-scores of 8.6 and 6.7, respectively.

As shown above, VexE prefers β-hydroxymyristoyl-ACP as the acyl chain donor. To localize VexE’s ACP-binding site, VexE from *S*. Typhi and ACP were cofolded using the open source AlphaFold implementation, ColabFold (43) (Fig. S5*C*). All five of the generated models showed a consistent ACP-binding mode, with ACP interacting predominantly with VexE α30. Analysis by PISA (44) indicates that the interface buries 616 Å^2^ of surface and has a favorable solvation free energy gain of 6.9 kcal/mol, as well as several favorable charge–charge interactions (where VexE positions three basic side chains to interact with four acidic ACP residues) (Fig. S5*D*). This interaction model places S^37^, the phosphopantetheine modification site in ACP, at the entrance to a narrow tunnel leading to the VexE active site. Significantly, PatA binds CoA using this same tunnel (17), and a superposition of PatA on VexE places the CoA β-phosphate immediately adjacent to the hydroxyl group of S^37^. We therefore modeled the phosphopantetheine group and myristoyl using S^37^ as a fixed point and the structure of the PatA myristoyl-CoA complex (PDB: 5f34) as a guide (Fig. 6*B*). In this model, the nonpolar butyryl moiety of the pantoic acid is positioned in a nonpolar pocket lined with V^534^, V^535^, and F^575^ and a favorable interaction between R^578^ and the phosphate. The phosphopantetheine group then extends through a narrow, predominantly nonpolar tunnel. The thioester carbonyl oxygen is positioned to make hydrogen bonds to the amide nitrogen and hydroxyl of S^510^, while the β-hydroxyl group sits in a more open pocket lined with hydrophilic residues, suggesting it may interact with bound water molecules. The β-hydroxymyristoyl group is positioned within a 15 Å deep, 4 to 6 Å wide tunnel lined on three sides by the highly curved central β-sheet and by α28 on the fourth. This tunnel is closed at its distal end by α27, and the myristoyl acyl chain occupies the full length of the tunnel; this argues that acyl chains longer than 14 carbon atoms would be excluded, while shorter acyl chains likely make fewer favorable interactions and therefore bind less strongly. This allows the tunnel to act as a molecular ruler, consistent with the observed substrate preference (Fig. 2*A*). Interestingly, PatA, which accommodates hexadecanoyl substrates (17), has a substrate-binding pocket that is noticeably wider and deeper than that of VexE.

With the acyl donor placed, attempts were made to model UDP-GlcNAc in the active site. However, the structure of VexE diverges considerably from PatA and LpxM in this region, so precedents were less useful. Instead, the known chemistry of the reaction was used to constrain the location of the O6 atom, and residue conservation was considered to guide the direction in which the ligand extends from the reaction site (Fig. 6*C*). In this model, O6 is placed within hydrogen bond distance of H^487^ (equivalent to the critical H^466^ in *A. denitrificans* VexE) and within 4 Å of the β-hydroxymyristoyl α-carbon. In turn, D^553^ (equivalent to D^532^ in *A. denitrificans* VexE) hydrogen bonds with both the backbone amide and Nδ nitrogen of H^487^. Note that these residues correspond to the His/Asp dyad previously identified as the key catalytic groups in PatA, as discussed above (17). The structural model suggests that S^510^ plays a secondary role in the reaction, serving as an oxyanion hole that stabilizes the partial negative charge developing on the thioester carbonyl through two hydrogen bonds. Other interactions stabilizing the GlcNAc group include hydrogen bonds between the N2 acetyl O and N^441^ and S^644^ and between O3 and O4 and R^356^. This latter interaction possibly helps rationalize the ability of VexE to discriminate against UDP-GalNAc, which is the C4 epimer of UDP-GlcNAc. The phosphate groups were positioned to form hydrogen bonds with G^645^ amide and the R^410^ and R^649^ guanidinium groups, while the uracil ring stacks on P^407^ and hydrogen bonds with N^405^ carbonyl oxygen and side chain. Note that the lack of good precedents and inherent uncertainties of side-chain packing in AlphaFold models means we are less confident overall in the details of the UDP-GlcNAc substrate placement, especially the nucleoside region, which makes relatively few contacts in our model.

### TPR domain structure and role

Searching the TPR domain using DALI suggests distant resemblance to a variety of TPR-repeat proteins from both bacterial and eukaryotic sources (Table S1). The closest homologs include the magnetosome protein MamA (PDB: 4xio; Z-score 17.8) and YbbR from *Bacillus subtilis* (PDB: 2q7f, Z-score 17.0), where both contain a region resembling the last five TPR repeats of VexE. Human Pim-1 has a similar Z-score (PDB: 3r02, Z-score 17.0), but the resemblance extends to the last eight repeats. Overall, this similarity seems to indicate a generic similarity between TPR domains with limited sequence identity (∼15%) but offers little by way of specific biological insight.

The N-terminal TPR domain comprises almost 60% of the VexE protein but does not seem positioned to interact with the substrates or otherwise assist directly with turnover, although the TPR domain does extend close enough the LPLAT active site that interactions with the nucleotide of UDP-GlcNAc are at least in principle possible. The interior face of the superhelix is more conserved than the exterior and is strongly basic, with a series of conserved arginine and lysine residues located predominantly at the N-terminal ends of these repeat helices (Fig. 5, *D* and *E*). The exposed central regions of these helices are generally conserved as small residues that are generally nonpolar or sometimes weakly polar. The net result is a shallow hydrophobic groove, flanked on one side by basic residues, that extends along the concave side of the TPR superhelix from the active site to around α7 in the TPR domain. We suggest that this domain may act as a product channel, allowing the product to leave the highly enclosed active site by sliding the acyl chain along the continuous nonpolar groove while the phosphate groups form a series of favorable charge–charge interactions with the ladder of conserved basic residues. Once the product reaches the exposed surface at the distal end of the TPR superhelix, it would be exposed for either transfer to the next enzyme in the pathway or to the cellular membrane.

## Discussion

The reported diacyl-HexNAc terminus in native Vi-antigen is reminiscent of intermediates of lipid A biosynthesis, and the sequence similarity shared by VexE and LpxM led to the initial proposal of a hypothetical pathway, where VexE was a secondary acyltransferase that might β-hydroxyacylate the UDP-β-hydroxymyristoyl-GlcNAc intermediate from the conserved lipid A-biosynthesis pathway (6). However, this proposal is not supported by the biochemical activity reported here, which clearly establishes VexE as a primary β-hydroxyacyl-ACP–dependent acyltransferase that acylates UDP-GlcNAc at C-6 to form UDP-6-*O*-[β-hydroxymyristoyl]-α-d-GlcNAc. Although the fragmentation of the native glycolipid terminus (6) was not fully informative, retrospective examination in the light of established VexE activity indicates C6-acylation can be accommodated by the previous mass spectrometry data. The novel activity of VexE reinforces the unique structure of the Vi antigen glycolipid terminus.

The availability of AlphaFold makes it possible to gain considerable insight into the working of proteins which are otherwise structurally uncharacterized, especially if (as in the case of VexE) there are other members of the family that are fairly well understood. In the case of VexE, a useful analog can be found in PatA, which participates in biosynthesis of the abundant mycobacterial PIMs by acylating a mannose residue at C-6 of the di- or tri-mannose intermediates (PIM_1_ or PIM_2_) (21). PatA belongs to the glycerol-3-phosphate acyltransferase clade of LPLAT proteins. While it shares very little sequence similarity with VexE, a key catalytic His-Asp/Glu dyad is conserved (16, 17, 18, 19) and modeling suggests VexE follows a similar catalytic mechanism with VexE H^466^ and D^532^ (*Achromobacter* numbering) being absolutely required for activity. An AlphaFold model of *S.* Typhi VexE suggests that ACP binds at the entrance to the substrate tunnel, and modeling indicates that the donor acyl chain inserts into a deep tunnel under the helix α28, as seen in PatA. The experimentally observed preference for 14 > 12 > 10 carbon-long acyl chains (but not 16) and the close fit of a 14 carbon chain in our model are consistent with this feature of VexE acting as a substrate ‘ruler’, similar to those seen in LpxM (16) and PatA (17, 18, 21). The absolute requirement for β-hydroxylated acyl donors is consistent with the mass spectrometry data for the terminal glycolipid, though the structure suggests that recognition of the hydroxyl group is likely indirect. The preference for UDP-GlcNAc supports the conclusion that the HexNAc residue in the VexE product has the *gluco* configuration. The GlcNAc acceptor–binding mode is less well constrained by available precedents, but modeling indicates that this substrate can be reasonably accommodated within the active site, so as to position O6 for an interaction with H^466^ and the thioester group. The N-terminal TPR domain of VexE is a unique feature of this protein. Based on the presence of a long nonpolar groove flanked by conserved basic residues, we propose that it serves as a product channel to allow the product to migrate to a site distal from the active site without requiring an energetically unfavorable dissociation from the protein as a whole.

The identification of VexE as a primary acyltransferase leaves questions about the origin of the second acyl chain observed in the natural product. VexE is the only dedicated acyltransferase encoded by the Vi antigen-biosynthesis genetic locus. Earlier, it was proposed that this additional acyltransferase could be provided by the conserved lipid A biosynthesis enzymes (6). Lipid A acyltransferases have exquisite specificity for acyl chain length and substitution position (27, 45, 46, 47). Attempts to generate a diacyl derivative with LpxA (which generates UDP-3-*O*-[β-hydroxyacyl]-GlcNAc (36)) and VexE were not successful (Fig. S6) but the negative results may simply reflect limitations of the conditions in our *in vitro* reactions. VexE demonstrates relatively low reaction efficiency *in vitro*. The same is true for LpxA. UDP-GlcNAc is required for producing essential glycoconjugates including lipid A (20) and peptidoglycan (48) and the equilibrium of the LpxA reaction favors the substrates (36). The reaction is driven forward by product consumption in the next step by the LpxC *N*-acetylesterase, and the amount of LpxC is highly regulated according to LPS requirement (49). Similarly, the VexE reaction may be driven forward either by the second acylation reaction or addition of the next sugar residue in the glycan backbone. Clearly, the enzyme responsible for adding the second acyl chain will only be resolved by extensive further research on these enzymes and their physical requirements, including the possible incorporation of membrane lipids and detergents.

The sequence of reactions in biosynthesis of the Vi antigen remain to be resolved but the identification of the precise activity of VexE, and discovery of a novel glycolipid structure in Gram-negative bacteria, provides the vital first step and a foundation for these analyses. While most bacterial polysaccharides involve addition of glycose residues to the nonreducing terminus of an acceptor, the bacterial class I hyaluronan synthases indicate that growth at the reducing end is also possible (25, 26, 50). In the context of Vi antigen biosynthesis, the acylated GlcNAc may therefore provide an acceptor on which the polysaccharide chain is extended, or a terminating molecule to which the chain is transferred to block any further elongation. Since a *vexE*-deletion mutant can still polymerize and export Vi antigen (6), both remain formally possible. In either scenario, the Vi polymerizing GT (TviE) must be able to bind two donors; UDP-GalNAcA and (acylated) UDP-GlcNAc. Although not strictly comparable, hyaluronan synthase requires two donor substrates (UDP-GlcA and UDP-GlcNAc) (24). The inherent gelling properties of Vi antigen polysaccharide may create challenges for the cellular machinery involved in its production. In WT bacteria, nascent Vi antigen is protected from enzymatic degradation during synthesis and export, suggesting close proximity of the synthesis machinery and cognate ABC transporter, as well as temporal coupling of the processes (51). The O-acetyltransferase TviD associates with the membrane and possesses a C-terminal TPR region that recruits TviE (22). The Vi antigen assembly system therefore offers a prototype to understand important fundamental principles in bacterial glycoconjugate assembly.

## Experimental procedures

### Bacterial strains and growth conditions

Bacteria were grown in LB broth containing ampicillin (100 μg/ml) or kanamycin (50 μg/ml) as required. The bacterial strains and plasmids are described in Table 3.

**Table 3:** Bacterial strains and plasmids used in this study

Strain or plasmid	Genotype or property	Source or reference
*Escherichia coli*		
Top10	F^−^*mcr*A Δ(*mrr*-*hsdRMS*-*mcrBC*) φ80 *lac*ZΔM15 Δ*lacX74 deoR nupG recA1 araD139 Δ*(*ara-leu*)7697 *galU galK rpsL*(Str^r^) *endA1*	Invitrogen
BL21 (DE3)	B F^−^*ompT gal dcm lon hsdS*_*B*_(*r*_*B*_^−^*m*_*B*_^−^) λ(DE3 [*lacI lacUV5*-*T7p07 ind1 sam7 nin5*]) [*malB*^+^]_K-12_(λ^S^)	Invitrogen
*Salmonella enterica*		
H251.1	*S. enterica* serovar Typhi Ty2 *trp cys* Δ*aroC*1019	(59)
CWG1236	*S. enterica* serovar Typhi Ty2 (strain H251.1) derivative Typhi Ty2 *trp cys* Δ*aroC*1019 Δ*vexE*	(6)
*Plasmid*		
pBAD24	Plasmid vector with L-arabinose-inducible promoter; Ap^r^	(60)
pET28a(+)	Plasmid vector with IPTG-inducible promoter; Km^r^	Novagen
pWQ787	pBAD24 derivative encoding *Achromobacter denitrificans* VexE-His_6_; Ap^r^	(6)
pWQ789	pBAD24 derivative encoding *Achromobacter denitrificans* VexE^H466A^-His_6_; Ap^r^	(6)
pWQ892	pET28a(+) derivative encoding *E. coli* ACP and ACPS; Km^r^	(31) This Study
pWQ890	pBAD24 derivative encoding *Vibrio harveyi* AasS-His_6_; Ap^r^	This Study
pWQ893	pWQ787 derivative encoding *Achromobacter denitrificans* VexE^D532A^-His_6_; Ap^r^	This Study
pWQ894	pWQ789 derivative encoding *Achromobacter denitrificans* VexE^H466AD532A^-His_6_; Ap^r^	This Study

### DNA methods

Oligonucleotide primers were obtained from Sigma-Aldrich and are described in Table S2. DNA fragments were PCR-amplified employing primers that introduced restriction sites for use in cloning. PCR products were digested using appropriate restriction enzymes (NEB; Invitrogen) and ligated to digested vector DNA using T4 DNA ligase (NEB). Plasmid pWQ892, which encodes both ACP-His_6_ and ACPS cloned from *E. coli* W3110, was generated by replicating a previously published method (31). Site-directed mutations in cloned genes were generated using KOD HotStart DNA polymerase (Novagen) with complementary primers containing desired point mutations (described in Table S2), by the QuikChange method (Stratagene). *Vibrio harveyii AasS* was codon optimized for expression in *E. coli* and synthesized by GeneArt (Thermo Fisher Scientific). Plasmids and PCR products were purified using the PureLink Quick Plasmid Miniprep Kit and PureLink PCR Purification Kit (ThermoFisher), respectively, according to the manufacturers’ instructions. All DNA constructs were confirmed by Sanger sequencing at the Genomics Facility, Advanced Analysis Centre, University of Guelph.

#### Bioinformatic analyses and structural modeling

Putative protein domains were identified using BLAST (52), the conserved domain database (53). Multiple sequence alignments were generated using Clustal Omega (54) and presented using ESPript (55).

For structural modeling, AF-P43112-F1-model_v2 was retrieved from the EBI database. The structure of *S.* Typhi VexE in complex with ACP was generated using ColabFold, using single copies of the sequences UNIprot P43112 (VexE) and P0A6B2 (ACP) as input. Note that while the AlphaFold *Achromobacter* VexE model (AF-A0A160EBU3-F1) has similar confidence scores, the acyl-binding tunnel is occluded by a rotation and repositioning of α28. ColabFold produces a similar defect, suggesting some subtlety of the *Achromobacter* sequence leads to a slight mispacking of this region that narrows the tunnel below the diameter of the solvent probe used to define molecular surfaces. The β-hydroxymyristoyl-phosphopantetheine ligand was modeled manually in Pymol, using the structure of myristoyl-CoA bound PatA (PDB: 5f34) as a guide. The complex was then refined using Rosetta minimization. UDP-GlcNAc was placed manually within the refined β-hydroxymyristoyl-phosphopantetheine model and refined using Rosetta minimization.

#### Purification of VexE and mutant derivatives

An overnight culture of *E. coli* Top10 transformed with pWQ787 was grown in LB supplemented with 100 μg/ml ampicillin and used to inoculate (1:100) 6 l of fresh medium. The culture was grown at 37 °C. When the culture A_600_ reached 0.6, L-arabinose was added to 0.02% (w/v) to induce recombinant protein expression and growth was continued for 4 h. Cells were collected by centrifugation at 5000*g* for 20 min and stored at −80 °C. The cell pellet was resuspended in 200 ml buffer A (125 mM Na-Hepes, 30% (w/v) glycerol, pH 7.5), supplemented with four cOmplete protease inhibitor tablets (Roche). Cells were lysed by passing the cell suspension through an Emulsiflex homogenizer (Avestin) at 15,000 psi. Unbroken cells and membranes were removed by centrifugation at 100,000*g*, for 1 h, at 4 °C. Four milliliters of preequilibrated Ni^2+^-NTA agarose resin (Qiagen) were added to the clarified lysate, and the mixture was incubated on a nutator for 1 h at 4 °C before loading into a gravity flow column. The column was washed with 10 column volumes of buffer A supplemented with 20 mM imidazole. VexE-His_6_ eluted in five column volumes of buffer A supplemented with 300 mM imidazole. The eluate was dialyzed into buffer B (100 mM Hepes, 50 mM L-arginine, 10% (w/v) glycerol, pH 7.5) using a 3.5 kDa MWCO dialysis membrane (Spectrum Labs), at 4 °C. VexE-His_6_ was concentrated to 2 ml using a 30 kDa MWCO centrifugal concentrator (Vivaspin 20, Sartorius). VexE-His_6_ was then further purified by gel filtration chromatography in buffer B employing an AKTA Pure FPLC equipped with a HiPrep 16/60 Sephacryl-S200 High Resolution gel filtration column. Elution was monitored by absorbance (280 nm), fractions containing VexE-His_6_ were confirmed by SDS-PAGE, pooled, and concentrated using a 30 kDa MWCO centrifugal concentrator (Vivaspin 20, Sartorius). The yield was ∼25 mg (50 mg/ml). Protein was stored at −80 °C in buffer B. VexE-His_6_ concentration was estimated based on the theoretical extinction coefficient at 280 nm of 94,100 M^−1^ cm^−1^ (ProtParam (56)).

#### Purification of holo-acyl carrier protein

Six liters of LB were supplemented with 50 μg/ml kanamycin and inoculated at 1:100 with an overnight culture of *E. coli* BL21(DE3) transformed with pWQ892. Cultures were grown with 200 rpm shaking at 37 °C, until A_600_ reached 0.6. Protein expression was then induced by addition of 1 mM (final concentration) IPTG and growth was continued for 6 h at 30 °C. Cells were collected by centrifugation for 20 min at 5000*g* and stored at −80 °C. Cells were resuspended in 100 ml buffer C (20 mM Na-Hepes, 200 mM NaCl, 2 mM DTT, 10% (w/v) glycerol, pH 8.0) and lysed by passing the suspension through an Emulsiflex homogenizer (Avestin) at 15,000 psi. The lysate was clarified by centrifugation for 1 h at 100,000*g*, 4 °C. The supernatant was applied by gravity flow to a 3 ml Ni^2+^-NTA agarose column (Qiagen) that was preequilibrated with buffer C. The column was washed with 10 column volumes of buffer C supplemented with 20 mM imidazole. Holo-ACP-His_6_ coeluted with holo-ACP synthase in five column volumes of buffer C supplemented with 250 mM imidazole. The eluate was dialyzed into buffer D (25 mM MOPS, 50 mM NaCl, 2 mM DTT, pH 7.5) using a 3.5 kDa MWCO dialysis membrane (Spectrum Labs) and diluted to 50 ml in buffer D. Holo-ACP-His_6_ was separated from holo-ACP synthase by ion exchange chromatography on an Akta Pure FPLC equipped with a 5 ml HiTrap QFF column (GE healthcare). The column was preequilibrated with 10 column volumes of buffer D. The dialysate was then applied at a flow rate of 1 ml/min; flow rate was otherwise maintained at 5 ml/min. The column was washed with 10 column volumes of buffer D. Elution occurred over a 20-column volume gradient of 0 to 50% buffer E (25 mM MOPS, 1 M NaCl, 2 mM DTT, pH 7.5). Fractions containing holo-ACP were monitored by absorbance at 215 nm and confirmed by SDS-PAGE. These fractions were pooled, dialyzed into buffer F (25 mM MOPS, 300 mM NaCl, pH 7.5) using a 3.5 kDa MWCO dialysis membrane (Spectrum Labs), then diluted to 50 ml in buffer F. Four milliliters of preequilibrated thiopropyl-sepharose 6B (GE Healthcare) was added and incubated with mixing on a nutator at 4 °C for 16 h. The resin was collected in a gravity flow column and washed with 10 column volumes of buffer F. Holo-ACP-His_6_ eluted in five column volumes of buffer F supplemented with 1 mM EDTA and 25 mM DTT. The eluate was concentrated using a 3 kDa MWCO centrifugal concentrator (Vivaspin20, Sartorius), exchanged into buffer F using a PD10 desalting column (GE Healthcare), and stored at −80 °C. Holo-ACP-His_6_ concentration was estimated based on the theoretical extinction coefficient at 280 nm of 1490 M^−1^ cm^−1^ (ProtParam (56)).

#### Purification of acyl-ACP synthetase

One liter of LB was supplemented with 100 μg/ml ampicillin and inoculated at 1:100 from an overnight culture of *E. coli* Top10 harboring pWQ890, which encodes C-terminally hexahistidine-tagged acyl-ACP synthetase (AasS) from *V. harveyi* (31, 32). Cultures were grown with 200 rpm shaking at 37 °C, until A_600_ reached 0.5. Recombinant protein expression was induced by addition of 0.02% (w/v) L-arabinose and growth was continued for 16 h at 20 °C. Cells were collected by centrifugation for 20 min at 5000*g*, and stored at −80 °C. Cells were then resuspended in 25 ml buffer G (20 mM Tris–HCl, 350 mM NaCl, 10 mM imidazole, pH 7.5), supplemented with a cOmplete mini protease inhibitor tablet, 20 μg/ml RNase A, and 20 μg/ml DNase I (Roche), and lysed by passing the suspension through a French Pressure Cell at 12,000 psi. Unbroken cells were removed by centrifugation for 20 min at 4000*g*, 4 °C, and membranes were removed by centrifugation for 1 h at 100,000*g*, 4 °C. Two milliliters of preequilibrated Ni^2+^-NTA agarose (Qiagen) were added to the supernatant, which was then incubated with mixing on a nutator for 1 h at 4 °C. The resin was collected in a gravity flow column then washed with 10 CV buffer G supplemented with 20 mM imidazole. AasS-His_6_ was eluted in 5 CV buffer G supplemented with 250 mM imidazole. The eluate was dialyzed into buffer H (20 mM Tris–HCl, 10% (w/v) glycerol, 1 mM EDTA, 100 μM DTT, 0.002% (v/v) Triton X-100, pH 7.5), using a 3.5 kDa MWCO dialysis membrane (Spectrum Labs). AasS-His_6_ was concentrated to 7 mg/ml using a 30 kDa MWCO centrifugal concentrator (Vivaspin20, Sartorius) and stored at −80 °C. AasS-His_6_ concentration was estimated based on the theoretical extinction coefficient at 280 nm of 66,030 M^−1^ cm^−1^ (ProtParam (56)).

#### Generation of acyl-acyl carrier protein donors

ACP acylation reactions contained 100 μM purified holo-ACP, 0.5 μM purified *V. harveyi* AasS, 300 μM fatty acid (Sigma; stocked in 100% ethanol), 100 mM Tris–HCl, pH 7.5, 10 mM ATP, and 1 mM MgCl_2_. The reaction volume was 1 ml. Reactions were incubated at 37 °C for 1 h, then concentrated using a 3 kDa MWCO centrifugal concentrator (Vivaspin20, Sartorius). Reaction progress was monitored by SDS-free tris-glycine PAGE in 2.5 M urea at pH 9.5 (33) and typically occurred to completion. Acyl-ACPs were purified and exchanged into buffer J (25 mM MOPS, 300 mM NaCl, pH 7.5) by gel filtration, at 0.5 ml/min, using an AKTA Pure FPLC equipped with a HiPrep 16/60 Sephacryl-S200 High Resolution column (GE Lifesciences). Fractions containing acyl-ACPs were identified by absorbance at 215 nm and confirmed by SDS-PAGE. Acyl-ACPs were concentrated to ∼20 mg/ml and stored at −80 °C. Acyl-ACP concentrations were estimated using a theoretical extinction coefficient at 280 nm of 1490 M^−1^ cm^−1^ (ProtParam (56)).

#### VexE acyltransferase assay

VexE acylation reactions were routinely performed in 20 μl volumes and contained 45 μM UDP-[1-^14^C]GlcNAc (ARC0151; 55 mCi/mmol, 0.1 mCi/ml), 100 μM purified acyl-ACP, 10 μM purified VexE, and 100 mM Na-Hepes, pH 7.5. Reactions were incubated at 25 °C for 16 h and analyzed by TLC. For TLC, 1.5 μl aliquots of reaction mixture were spotted and dried on TLC plates (Fluka Analytical; silica gel on Al foil, 60 Å medium pore diameter). TLC plates were developed in ethyl acetate-butanol-glacial acetic acid-water (10:10:8:5). After drying, the plates were exposed on phosphor storage screens (Kodak) for 2 days, then imaged using a Personal FX Phosphor Imager (Bio-Rad).

HPLC analysis of VexE activity was performed on an Agilent 1200 high performance liquid chromatograph interfaced with an Agilent UHD 6530 Q-ToF mass spectrometer. An Agilent C18 column (Poroshell 120, EC-C18, 50 mm × 3.0 mm, 2.7 μm) was used for separation. The mobile phase consisted of solvent A (0.1% (v/v) formic acid in H_2_O) and solvent B (0.1% (v/v) formic acid in acetonitrile). The mobile phase program was as follows: 1 min 10% B, increase to 100% B over 29 min, 5 min 100% B, and 20 min reequilibration. Flow rate was 0.4 ml/min. The electrospray capillary voltage was 4.0 kV. Nitrogen was the drying gas (250 °C, 8 l/min) and the nebulizing gas (30 psi). The fragmentor was set to 160 V. The mass-to-charge ratio was scanned in negative ion mode over 100 to 3000 *m*/*z* (2 GHz extended dynamic range). Extracted ion chromatograms were generated using a mass accuracy of 0.015 *m*/*z*. Acquisition rate was 2 spectra/s. ESI TuneMix (Agilent) was used for calibration. Injection volume was 2 μl. Data analysis employed Qualitative Analysis software (Agilent).

#### Characterization of VexE reaction product

To isolate sufficient product for structural determination, VexE acylation reactions were scaled up to 20 ml and contained 10 mM UDP-GlcNAc, 100 μM holo-ACP, 0.5 μM AasS, 25 μM VexE, 300 μM β-hydroxymyristate, 10 mM ATP, 1 mM MgCl_2_, and 100 mM Na-Hepes, pH 7.5. The reaction mixture was incubated at 25 °C for 2 h. Protein was removed using a 3 kDa MWCO centrifugal concentrator (Vivaspin20, Sartorius); the flow-through was collected and concentrated to 1 ml using a SpeedVac vacuum concentrator (ThermoFisher Scientific). This sample was then purified by HPLC, employing an Agilent 1260 Infinity II chromatography system, equipped with a semi-preparative reverse-phase liquid chromatography column (Phenomenex 250 × 10 mm, Synergi 4 μm Fusion-RP 80 Å). Mobile phases were H_2_O (A) and acetonitrile (B). Flow rate was 3.4 ml/min. The mobile phase program was 2% B for 5 min, increase to 80% B over 20 min, hold 80% B for 5 min, then reequilibrate at 2% B for 10 min. Injection volume was 100 μl. Elution was monitored by absorbance at 254 nm (Agilent VWD 1260). The presence of β-hydroxymyristoyl-UDP-GlcNAc was confirmed by MS, by manual infusion into a Bruker AmaZon SL ion trap mass spectrometer at the Advanced Analysis Centre Mass Spectrometry Facility (University of Guelph). Electrospray capillary entrance and exit voltages were set to 4 kV and 140 V, respectively. Nitrogen was used as the drying gas, which was supplied at 300 °C. The mass-to-charge ratio was scanned across the range of 500 to 1000 *m*/*z* in negative-ion mode. Data were analyzed using Bruker DataAnalysis 4.2 (https://www.bruker.com/en/products-and-solutions/mass-spectrometry/ms-software.html). Fractions were dried using a SpeedVac vacuum concentrator (ThermoFisher Scientific). ∼0.5 mg HPLC-purified VexE product was deuterium exchanged by lyophilizing twice from 99.9% D_2_O and examined in a solution of 99.96% D_2_O at 25 °C. ^1^H and ^13^C NMR spectra were collected on a 600 MHz Bruker AVANCE III spectrometer equipped with a 5 mm TCI cryoprobe, and ^31^P spectra were collected using a Bruker 400 MHz Avance III spectrometer equipped with a 5 mm broadband Prodigy cryoprobe both located in the Advanced Analysis Centre NMR Facility. Data were analyzed using Bruker TopSpin software (https://www.bruker.com/en/products-and-solutions/mr/nmr-software/topspin.html). Sodium 3-trimethylsilylpropanoate-2,2,3,3-*d*_4_ was used as an internal standard (δ_H_ 0, δ_C_ −1.6). Referencing of ^31^P spectra was performed by substitution with a solution of 85% phosphoric acid (δ_P_ 0 ppm).

#### PAGE and immunoblotting

To examine proteins, whole-cell lysates were prepared by suspending 1 A_600_ unit-equivalent of cells in 100 μl SDS-PAGE buffer (57). SDS-PAGE samples were incubated at 100 °C for 10 min, prior to electrophoresis (tris-glycine, 10% (w/v) acrylamide). Proteins were stained with Coomassie Brilliant Blue R-250. For immunoblotting, protein samples were transferred to nitrocellulose membranes (Amersham Protran, 0.45 μm). Primary antibodies were murine monoclonal anti-His_5_ (Qiagen; diluted 1:3000) and secondary antibodies were either horseradish peroxidase–conjugated goat anti-mouse IgG (Qiagen; diluted 1:3000). Detection employed horseradish peroxidase–substrate Luminata Classico (Millipore). To analyze polysaccharides in whole-cell lysates, samples were prepared as above and then incubated with 50 μg proteinase K for 1 h at 55 °C. The lysates were then separated by SDS-PAGE and transferred to PVDF (Amersham HyBond P 0.45 μm) or nylon membranes (BioDyne B; Pall). Membranes were probed with murine monoclonal antigen antibody P2B1G2/A9 ((58) diluted 1:350), followed by alkaline phosphatase–conjugated goat anti-mouse secondary antibody (Qiagen; diluted 1:3000). Colorimetric detection employed nitro-blue tetrazolium and 5-bromo-4-chloro-3-indolyl phosphate (Roche).

## Data availability

All data are contained in the article.

## Supporting information

This article contains supporting information.

## Conflict of interest

The authors declare that they have no conflicts of interest with the contents of this article.
