# Supplementary material for: A dedicated C-6 β-hydroxyacyltransferase required for biosynthesis of the glycolipid anchor for Vi antigen capsule in typhoidal Salmonella
Source: J Biol Chem. 2022 Sep 22;298(11):102520. doi: 10.1016/j.jbc.2022.102520 (PMC9597891; doi:10.1016/j.jbc.2022.102520)
Supplement: Supplemental Figures S1–S6 and Tables S1, S2 [file mmc1.pdf]

Supplementary Information

A dedicated C-6 β-hydroxyacyltransferase required for biosynthesis of the glycolipid anchor for Vi antigen capsule in typhoidal *Salmonella*.

Liston, S. D.[1,2], Ovchinnikova, O.G.[1], Kimber, M.S.[1] and Whitfield, C.[1,3]

[1]Department of Molecular and Cellular Biology, University of Guelph, Guelph, Ontario, Canada.

[2]Current address, Department of Molecular Genetics, University of Toronto, Toronto, Ontario, Canada.

[3]To whom correspondence should be addressed: Dept. of Molecular and Cellular Biology, University of Guelph, 50 Stone Road East, Guelph, Ontario N1G 2W1, Canada. Tel 1-519-824-4120 (ext 53361); email cwhitfie@uoguelph.ca.

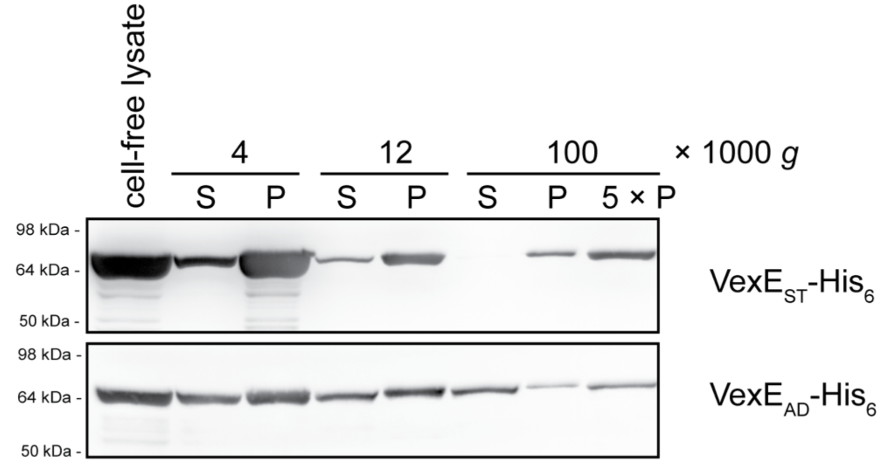

Supplementary Figure 1. Solubility of VexE proteins from different species. The figure shows western immunoblot data for fractionation of cell-free lysates of *E. coli* Top10 containing hexahistine-tagged homologs from *S*. Typhi (VexE$_{ST}$-His$_6$) and *A. denitrificans* ((VexE$_{AD}$-His$_6$). The soluble (S) and pellet (P) fractions were examined from the indicated differential centrifugation steps. In the final 100 000 ×*g* step, the S and P fractions represent the cytoplasm/periplasm and membrane components, respectively. 5 × P indicates loading of a 5-fold excess of the membranes. In both panels, the hexahistidine tag was detected using mouse anti-His$_5$ monoclonal antibody (Qiagen) diluted 1: 2000 and HRP-conjugated goat anti-mouse secondary antibody (Qiagen) diluted 1:3 000, coupled with the chemiluminescent substrate Luminata Classico (Millipore).

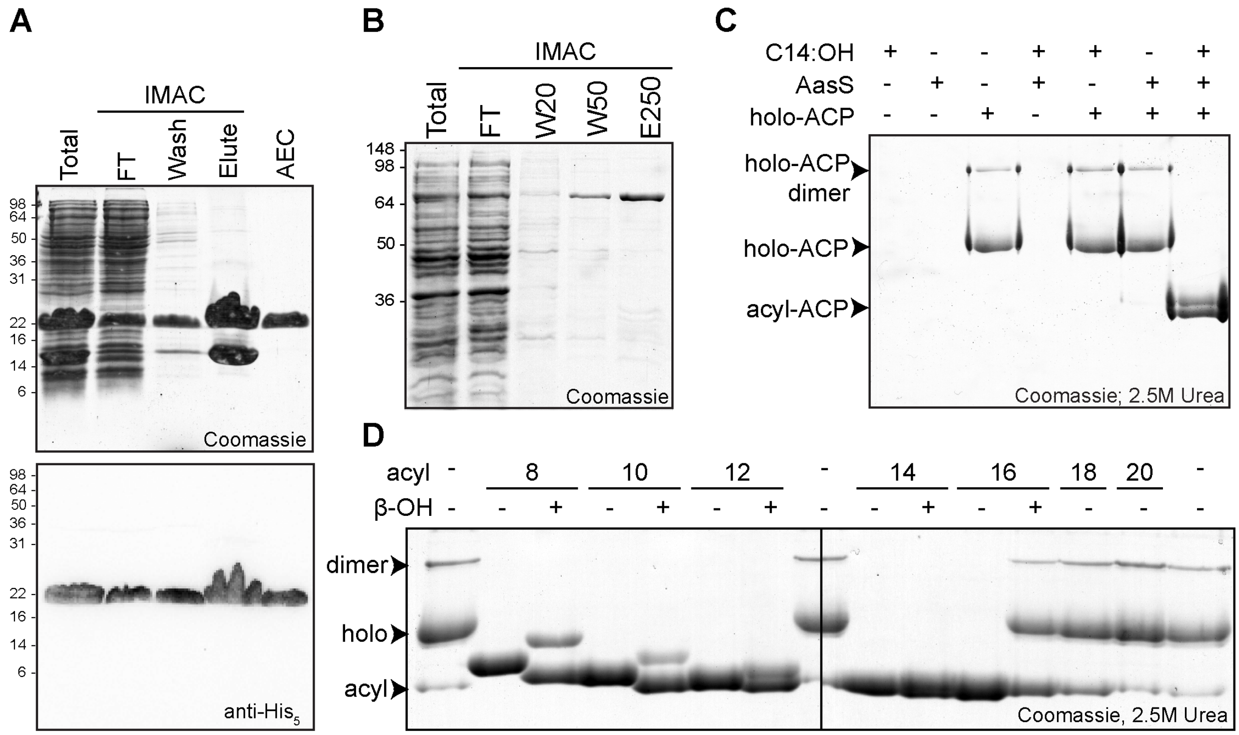

Supplementary Figure 2. Generation of acyl-ACP donors. (***A***) SDS-PAGE (*upper*) and the corresponding western immunoblot probed with His_5-specific antibody (*lower*) of fractions from the holo-ACP purification. ACP-His_6 and holo-ACP synthase (AcpS) were co-expressed and then co-purified from *E. coli* by immobilized metal-affinity chromatography (IMAC). AcpS was then removed by anion-exchange chromatography (AEC). Although the molecular weight of ACP-His_6 is lower than AcpS, it consistently shows lower relative mobility in SDS-PAGE. FT: flow-through (***B***) SDS-PAGE of fractions from the IMAC purification of *V. harveyi* AasS. (***C***) *V. harveyi* AasS specifically acylates holo-ACP *in vitro* with high-efficiency. The panel shows the results of SDS-free tris-glycine PAGE in 2.5 M urea at pH 9.5, stained with Coomassie Brilliant Blue R250. Acylation reactions contained DL-β-hydroxymyristic acid, purified AasS, and purified holo-ACP where indicated. The electrophoretic mobility of ACP increases when loaded with acyl-cargo, which only occurred when all reagents were included. (***D***) AasS effectively loads holo-ACP with fatty acids and their DL-β-hydroxylated derivatives from 8-16 carbons in chain length. Negative control reactions contained holo-ACP and AcpS incubated in reaction mixtures lacking any fatty acid substrate. The panel shows PAGE of acylation reactions prepared as in *B*. Carbon chain-length of the acyl-donors are indicated. The vertical line marks two separate gels.

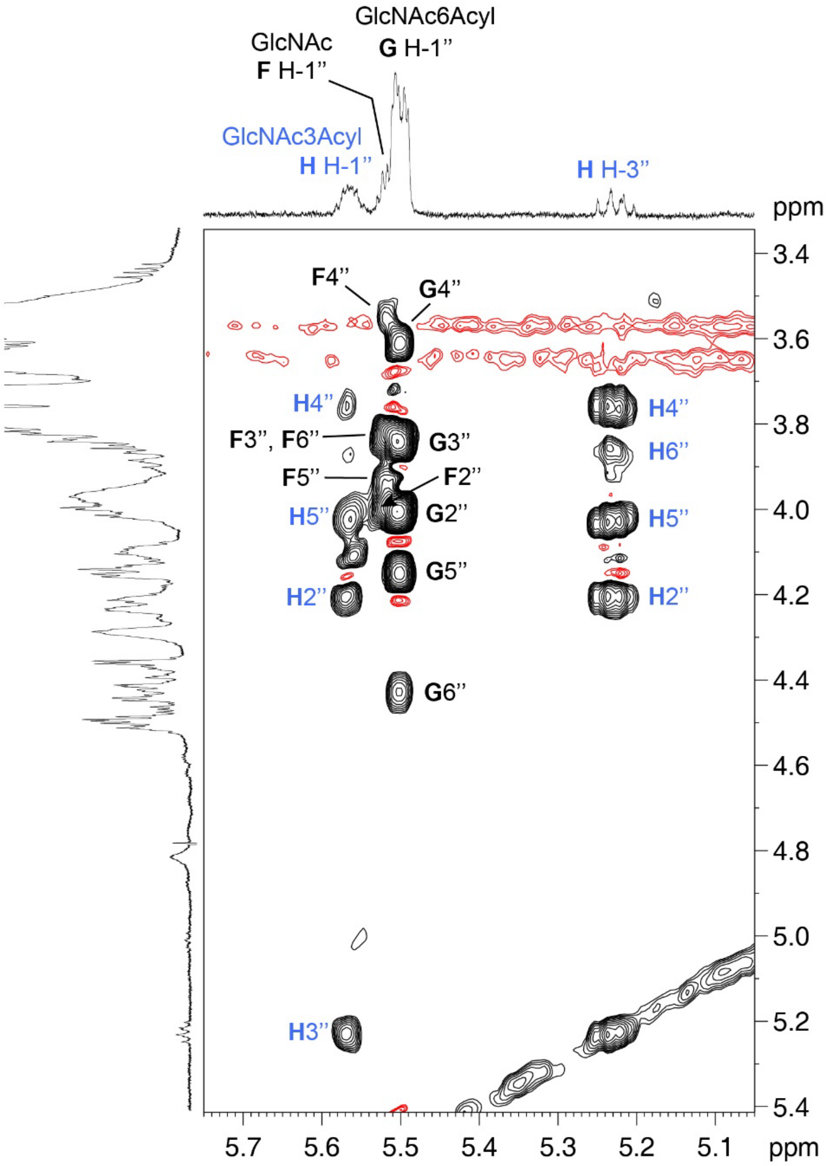

Supplementary Figure 3. Part of the ^{1}H,^{1}H TOCSY spectrum of VexE *in vitro* product demonstrating correlations from anomeric protons. The corresponding parts of ^{1}H NMR spectrum are shown along the horizontal and vertical axes. The signals for minor 3-acylated product (residue **H**) are marked in blue. The signal for **H** H-1 overlap with some other (unassigned) signal and thus appear to be higher intensity then **H** H-3.

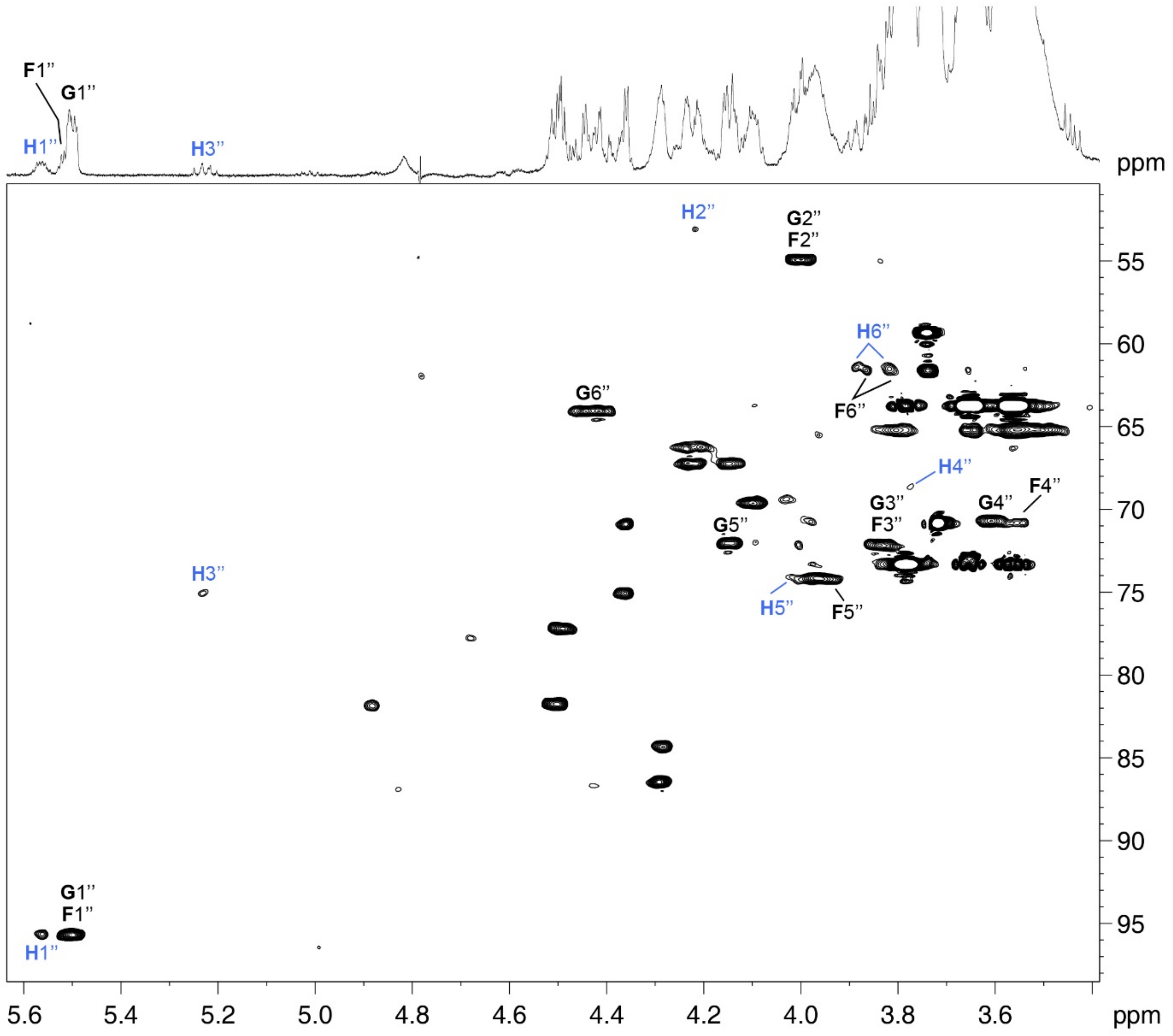

Supplementary Figure 4. Part of the ^{1}H,^{13}C HSQC spectrum of VexE *in vitro* product. Lower-level cut (compared to the HSQC spectrum shown in Figure 5) shows correlations from a minor product. C/H pairs of sugar residues are labelled as follows: **G**, GlcNAc6Acyl, **H**, GlcNAc3Acyl (marked in blue), **F**, unsubstituted GlcNAc. The corresponding part of ^{1}H NMR spectrum is shown along the horizontal axis.

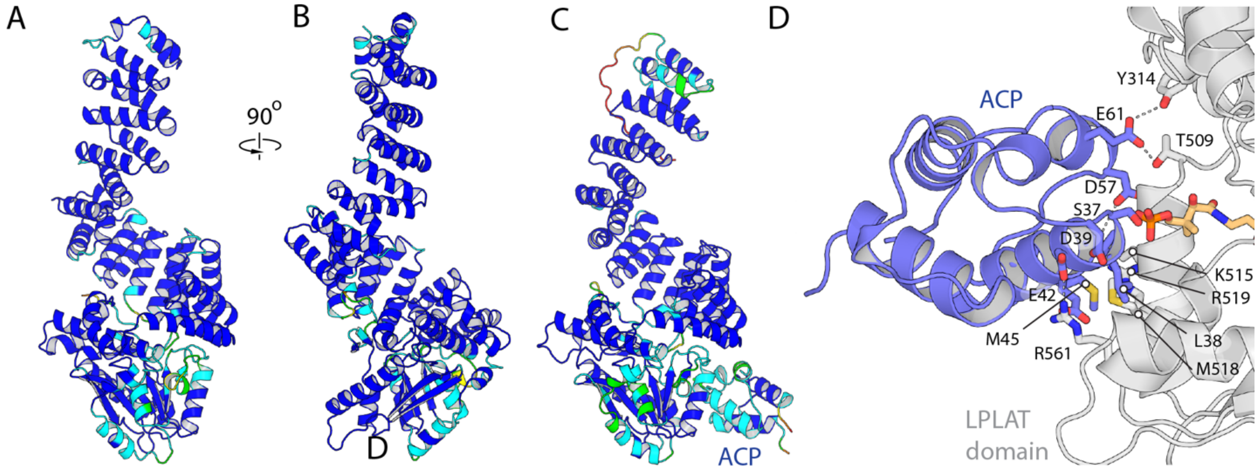

Supplementary Figure 5. Supplementary structural figures. (***A***) pLDDT score mapped onto the AlphaFold model for *S.* Typhi VexE. Regions with predicted local distance difference test score (pLDDT) greater than 90 are blue; pLDDT between 80 and 90 are shown in cyan; pLDDT between 70 and 80 are shown in green; pLDDT between 70 and 60 are shown in yellow; pLDDT between 50 and 60 are shown in orange; regions with pLDDT less than 50 are shown in red. (***B***) The orthogonal view of *S.* Typhi VexE. (***C***) pLDDT scores mapped onto the VexE:ACP complex model. (***D***) Details of the VexE:ACP Collabfold generated complex model. The modelled β-hydroxymyristoyl-phosphopantetheine ligand is shown for visual reference but was not part of the prediction.

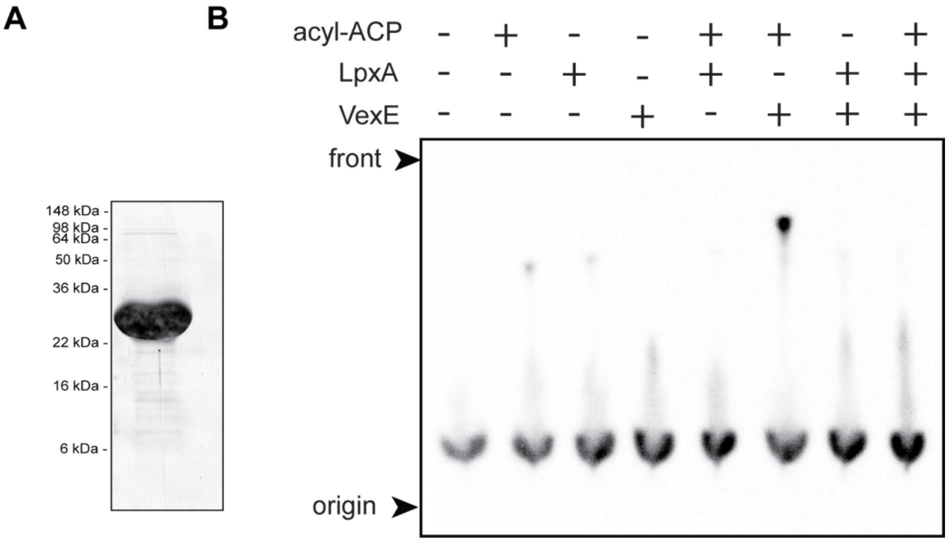

Supplementary Figure 6. Attempt to modify the VexE product using LpxA. (*A*) shows a Coomassie blue-stained PAGE gel with purified His_6-LpxA (from *E. coli* W3110). The open-reading frame encoding LpxA was amplified by PCR with the primers LpxA Forward and LpxA Reverse (**Supplementary Table 2**). The PCR fragment was cloned in pET28(+) as plasmid pWQ891. One liter of LB was supplemented with 50 µg/mL kanamycin and inoculated at 1:100 from an overnight culture of *E. coli* BL21(DE3) transformed with pWQ891. Cultures were grown with 200 rpm shaking at 37 °C, until OD_{600} reached 0.5. Recombinant protein expression was then induced by adding 1mM (final concentration) IPTG, and growth was continued for 16 h at 20 °C. Cells were collected by centrifugation for 20 min at 5 000 × *g*, and stored at $^{-}$80 °C. Cells were resuspended in 25 mL Lysis Buffer (20mM sodium phosphate, 350 mM NaCl, pH 7.0), supplemented with 1 cOmplete protease inhibitor tablet (Roche), 20 µg/mL RNase A, 20 µg/mL DNase I (Roche), and 10 mM imidazole. Cells were lysed by passing the suspension through a French Pressure Cell at 12 000 psi. Unbroken cells were removed by centrifugation for 20 min at 4 000 × *g*, 4 °C, and membranes were removed by centrifugation for 1 h at 100 000 × *g*, at 4 °C. Pre-equilibrated Ni^{2+}-NTA agarose (Qiagen, 2mL) was added to the supernatant, which was incubated on a nutator for 1 h at 4 °C. The resin was transferred to a gravity flow column and washed sequentially with 10 column volumes of Lysis Buffer supplemented with 20 and then 50 mM imidazole. His_6-LpxA was eluted in 5 column volumes of Lysis Buffer supplemented with 250 mM imidazole. The eluate was concentrated using a 3 kDa MWCO centrifugal concentrator (Vivaspin20; Sartorius), then further purified by gel filtration chromatography in Lysis Buffer

employing an AKTA Pure FPLC equipped with a HiPrep 16/60 Sephacryl-S200 High Resolution gel filtration column. Fractions containing His_6-LpxA were confirmed by SDS-PAGE, pooled, concentrated using a 3 kDa MWCO centrifugal concentrator (Vivaspin 20, Sartorius), and stored at $^-$80 °C. His_6-LpxA concentration was estimated based on the theoretical extinction coefficient at 280 nm of 9 190 $M^{-1}cm^{-1}$ (ProtParam). (***B***) shows a standard VexE assay supplemented by the addition of His_6- LpxA. The 20 µL reactions contained (as appropriate) 91 µM UDP-[1-^{14}C]GlcNAc (ARC0151; 55 mCi/mmol, 0.1 mCi/mL), 250 µM purified myristoyl-ACP, 25 µM purified VexE, 50 µM LpxA, 150 mM NaCl, and 50 mM Na-HEPES, pH 7.5. Note that inclusion of LpxA suppressed VexE activity and no new products were made. The reasons for this have not been resolved.

Supplementary Table 1. Top search hits from DALI using the *S.* typhi Alpha Fold LPLAT and TPR domains as search models.

**LPLAT (C-terminal) domain as search model**					
PDB	Z-score	r.m.s.d. (Å)	Length aligned	% identity	protein
5f2t	18.2	3.2	247	10	*M. smegmatis* PatA
5knk	17.7	3.4	233	15	*A. baumannii* LpxM
5kym	8.6	3.6	160	10	*T. maritima* PlsC
1k30	6.7	3.9	164	13	*C. moschata* GPA

**TPR (N-terminal) domain as search model**					
PDB	Z-score	r.m.s.d. (Å)	Length aligned	% identity	protein
4xi0	17.8	2.8	171	12	*D. magneticus* MamA
3vtx	17.5	2.2	170	15	*M. bavaricum* MamA
2q7f	17.0	2.6	178	15	*B. subtilis* YrrB
3ro2	17.0	3.4	263	15	*H. sapiens* Pim-1
6eou	15.8	3.1	193	17	*H. sapiens* OGT
4kxk	15.7	5.3	181	14	*H. sapiens* Pex5p
5a7d	15.0	3.3	228	13	*D. melanogaster* PINS

Supplementary Table 2. Sequences of oligonucleotide primers used to generate recombinant plasmids, site-directed mutants, and genomic deletions.

Primer	Sequence[a,b] (5′→3′)	Features
ACP-forward	gcgcgcgccatATGAGCACTATCGAAGAACGC	Forward primer for amplification of *E. coli acp*; introduces *Nde*1 site.
ACP-Reverse	TTTAAATTCTCCTTCCCGGGCCCGGTCACGCCTGGTGGCCGTT	Reverse primer for amplification of *E. coli acp*
ACPS-Forward	CCGGGCCCGGGAAGGAGAATTTAAAATGGCAATCTTAGGTTTAGGC	Forward primer for amplification of *E. coli acpS*.
ACPS-Reverse	gcgcgcgcctcgagTCAACTTTCAATAATTACCGT	Reverse Primer for amplification of *E coli acpS*, introduces *Xho*1 site.
SL113	cattCCatgggcagcagccaccatcaccatcatcacATGAACCAGTATGTAAATGATCC	Forward Primer for amplification of *E. coli aasS*; introduces sequence for a hexahistidine tag and *Nco1* site
SL114	cattaagcttttaCAGATGAAGTTTACGCAGTTC	Reverse primer for amplification of *E. coli aasS*; introduces *HindIII* site.
SL144	CTGTGCCTGGCCATCgccGGCGCGGCGAATCCCG	Forward mutagenesis primer for VexE D532A
SL145	CGGGATTCGCCGCGCCggcGATGGCCAGGCACAG	Reverse mutagenesis primer for VexE D532A
LpxA Forward	catttctagaAATAATTTTGTTTAACTTTAAGAAGGAGATATACCatgcaccatcaccatcatcacGTGATTGATAAATCCGCCTTTG	Forward primer for amplification of *E. coli lpxA*, introduces sequence for a hexahistidine tag and *Xba1* site
LpxA Reverse	cattaagcttttaACGAATCAGACCGCGCGTTG	Forward primer for amplification of *E. coli lpxA*, introduces *HindIII* site

[a]Restriction sites are underlined.

[b]Non-chromosomal sequences are lowercase.
